# Está na Hora da Angiotomografia de Coronárias ser Incorporada ao SUS

**DOI:** 10.36660/abc.20220033

**Published:** 2022-03-10

**Authors:** Nilson Tavares Poppi

**Affiliations:** 1 Instituto do Coração Hospital das Clínicas FMUSP São Paulo SP Brasil InCor - Instituto do Coração do Hospital das Clínicas da FMUSP, São Paulo, SP - Brasil

**Keywords:** Análise Custo-Benefício, Angina Estável, Doença da Artéria Coronariana, Angiografia por Tomografia Computadorizada, Sistema Único de Saúde (SUS, Diagnóstico por Imagem/métodos

Na avaliação dos pacientes com doença arterial coronária (DAC) estável, também chamada síndrome coronária crônica (SCC), os exames subsidiários são utilizados tanto para fins diagnósticos quanto prognósticos.^1 , 2^ Atualmente, temos disponíveis opções anatômicas (coronariografia e angiotomografia das coronárias) e funcionais (teste ergométrico, ecocardiograma com estresse, estudos de perfusão miocárdica de estresse e repouso por cintilografia, ressonância magnética e tomografia por emissão de pósitrons). A coronariografia, padrão-ouro, é considerada invasiva, sendo reservada para os pacientes clinicamente mais graves, ou com achados de pior prognóstico nos exames não invasivos, quando a revascularização miocárdica é considerada ou planejada.^3^

A escolha pelo método diagnóstico mais apropriado é uma etapa importante e desafiadora para o cardiologista na avaliação clínica em SCC. O primeiro passo para esta tomada de decisão, é o cálculo da probabilidade pré-teste (PPT) de DAC. Conforme recomendação das últimas diretrizes de SCC,^3 , 4^ os pacientes classificados como de alta PPT, devem receber o tratamento clínico medicamentoso e realizar exames para avaliação prognóstica. Na baixa PPT, a prioridade é iniciar pela busca de um diagnóstico alternativo mais provável do que DAC. Os pacientes com PPT calculada entre 15-85% estão na faixa intermediária, onde justamente os exames subsidiários são mais úteis e importantes para o diagnóstico.^5^ Além da acurácia diagnóstica e da PPT, a escolha ideal de um exame não invasivo depende das características clínicas dos pacientes, experiência local e disponibilidade dos testes.^4^ No Brasil, estima-se que até 80% da população dependa exclusivamente de atendimento médico pelo sistema público de saúde (SUS).^6^ Neste contexto de escassez e gerenciamento de recursos, médicos e gestores devem priorizar as opções mais custo-efetivas para o diagnóstico de DAC.

O artigo de Carmo et al.,^7^ analisa especificamente o panorama de PPT intermediária e intermediária-baixa (10-60%), através de dois métodos que analisam custo-efetividade, utilizando conceitos atuais de tecnologias em saúde (razão de custo-efetividade incremental e benefício líquido). Estratégias com testes sequenciais eram previstas quando o primeiro teste fosse positivo. Os resultados foram apresentados de acordo com a variação de PPT em diferentes limiares de disposição a pagar por um diagnóstico correto. Embora a angiotomografia coronariana (ATC) ainda não esteja disponível no SUS, foi a estratégia mais custo-efetiva neste estudo, isoladamente ou em exames sequenciais, exceto nas faixas mais baixas de disposição a pagar, quando foi superada pelo ecocardiograma de estresse (ECO).^7^

Um outro achado interessante diz respeito à utilização do Teste Ergométrico (TE), que embora tenha sido colocada em segundo plano em diretrizes internacionais,^4^ aparece como excelente opção de custo-efetividade em PPTs mais baixas e menores valores de disposição a pagar, principalmente quando complementado pelo ECO após um TE positivo.^7^ Diante das diferenças econômicas entre as regiões do Brasil, em localidades com menor disponibilidade de recursos e financiamento em saúde, o TE poderia se manter como principal estratégia de triagem diagnóstica de DAC.

A cintilografia miocárdica (CM), muito utilizada no SUS, se mostrou mais cara e menos efetiva do que a ATC e o ECO em todos os cenários avaliados, aparecendo como um destaque negativo na estratégia diagnóstica em DAC. Além disso, a ATC foi capaz de revelar DAC não obstrutiva mesmo em pacientes com isquemia miocárdica moderada e grave em exames funcionais (até 15% dos inicialmente selecionados para o estudo ISCHEMIA).^8^ Outra vantagem da ATC é a possibilidade de quantificação não invasiva da reserva de fluxo fracionada, capaz de detectar DAC obstrutiva com limitação de fluxo, reduzindo o número de exames com resultados falso-positivos.^9^ Tais achados reforçam a utilidade da ATC em poupar uma parcela considerável dos pacientes com SCC da realização do cateterismo, diminuindo adicionalmente os custos e efeitos adversos do exame invasivo.

Os principais resultados da análise realizada pelos autores são baseados na estimativa de custo da ATC no SUS, que pode estar subestimada, pois os valores pagos pela ATC e pela CM são mais próximos nos serviços de saúde complementar, o que representaria uma importante limitação deste estudo. Outra lacuna é o cenário de PPT intermediária-alta (60-85%), não avaliada neste trabalho, onde a CM poderia ser capaz de mostrar uma melhor competitividade, considerando seu bom desempenho em confirmar o diagnóstico de DAC funcionalmente significativa nesta faixa mais alta de PPT.^10^

Este artigo traz considerações relevantes que podem ser aplicadas por gestores e médicos do SUS na escolha pelo método diagnóstico de DAC, além de servir de referência para uma futura diretriz brasileira, que poderá considerar a recomendação da ATC como primeiro exame na avaliação de DAC, alternativamente aos exames funcionais, de forma análoga à outras diretrizes internacionais.^1 , 11^ Cabe salientar, entretanto, que as provas funcionais continuam sendo insubstituíveis quando se deseja avaliar objetivamente o grau de limitação funcional e a resposta terapêutica dos pacientes.^3 , 4^ Enfim, ainda há espaço para o uso racional de todos os métodos diagnósticos em DAC na prática clínica, mas já não há razão para a ATC não ser incorporada ao SUS.

## References

[1] Gottlieb I, Bittencourt MS, Rochitte CE, Cavalcante JL. Coronary Computed Tomography Angiography Takes the Center Stage and Here is Why. Arq Bras Cardiol. 2019;112(1):104-6. DOI: 10.5935/abc.201900 10.5935/abc.20190003 PMC6317632 30673022

[2] Newby DE, Adamson PD, Berry C, Boon NA, Dweck MR, Flather M, et al. Coronary CT Angiography and 5-Year Risk of Myocardial Infarction. N Engl J Med. 2018;379(10):924-33. doi: 10.1056/NEJMoa1805971. 10.1056/NEJMoa1805971 30145934

[3] Cesar LA, Ferreira JF, Armaganijan D, Gowdak LH, Mansur AP, Bodanese LC, et al. Guideline for stable coronary artery disease. Arq Bras Cardiol. 2014;103(2 Suppl 2):1-56. doi: 10.5935/abc.2014s004. 10.5935/abc.2014s004 25410086

[4] Knuuti J, Wijns W, Saraste A, Capodanno D, Barbato E, Funck-Brentano C, et al. 2019 ESC Guidelines for the diagnosis and management of chronic coronary syndromes. Eur Heart J. 2020;41(3):407-77. doi: 10.1093/eurheartj/ehz425. 10.1093/eurheartj/ehz425 31504439

[5] Fihn SD, Gardin JM, Abrams J, Berra K, Blankenship JC, Dallas AP, et al. 2012 ACCF/AHA/ACP/AATS/PCNA/SCAI/STS Guideline for the diagnosis and management of patients with stable ischemic heart disease: a report of the American College of Cardiology Foundation/American Heart Association Task Force on Practice Guidelines, and the American College of Physicians, American Association for Thoracic Surgery, Preventive Cardiovascular Nurses Association, Society for Cardiovascular Angiography and Interventions, and Society of Thoracic Surgeons. J Am Coll Cardiol. 2012;60(24):e44-e164. doi: 10.1016/j.jacc.2012.07.013 10.1016/j.jacc.2012.07.013 23182125

[6] Selig FA. Outlook and Perspectives in Diagnosis and Treatment of Congenital Heart Diseases in Brazil. Arq Bras Cardiol. 2020;115(6):1176-7. doi: 10.36660/abc.20200680. 10.36660/abc.20200680 PMC8133732 33470319

[7] Carmo PB, Magliano CAS, Rey HCV, Camargo GC, Trocado LFL, Gottlieb I. Análise da Custo-Efetividade da Angiotomografia Coronariana no SUS, em Comparação com Outros Métodos Não Invasivos na Suspeita de DAC Estável. Arq Bras Cardiol. 2022; 118(3):578-585. 10.36660/abc.20201050 PMC8959029 35137778

[8] Maron DJ, Hochman JS, Reynolds HR, Bangalore S, O’Brien SM, Boden WE, et al. Initial Invasive or Conservative Strategy for Stable Coronary Disease. N Engl J Med. 2020;382(15):1395-407. doi: 10.1056/NEJMoa1915922. 10.1056/NEJMoa1915922 PMC7263833 32227755

[9] Morais TC, Assunção-Jr AN, Dantas Júnior RN, Silva CFGD, Paula CB, Torres RA, et al. Diagnostic Performance of a Machine Learning-Based CT-Derived FFR in Detecting Flow-Limiting Stenosis. Arq Bras Cardiol. 2021;116(6):1091-8. doi: 10.36660/abc.20190329. 10.36660/abc.20190329 PMC8288523 34133592

[10] Knuuti J, Ballo H, Juarez-Orozco LE, Saraste A, Kolh P, Rutjes AWS, et al. The performance of non-invasive tests to rule-in and rule-out significant coronary artery stenosis in patients with stable angina: a meta-analysis focused on post-test disease probability. Eur Heart J. 2018;39(35):3322-30. doi: 10.1093/eurheartj/ehy267. 10.1093/eurheartj/ehy267 29850808

[11] Moss AJ, Williams MC, Newby DE, Nicol ED. The Updated NICE Guidelines: Cardiac CT as the First-Line Test for Coronary Artery Disease. Curr Cardiovasc Imaging Rep. 2017;10(5):15. doi: 10.1007/s12410-017-9412-6. 10.1007/s12410-017-9412-6 PMC5368205 28446943

