# Supplementary material for: Está na Hora da Angiotomografia de Coronárias ser Incorporada ao SUS
Source: Arq Bras Cardiol. 2022 Mar 10;118(3):586–7. [Article in Portuguese] doi: 10.36660/abc.20220033 (PMC8959036; doi:10.36660/abc.20220033)
Supplement: Translation

# Translation: It is Time for Coronary Computed Tomography Angiography to be Incorporated into the SUS

**Authors:** Nilson Tavares Poppi

**Affiliations:** 1 Brazil InCor - Instituto do Coração do Hospital das Clínicas da FMUSP, São Paulo, SP - Brazil

**Keywords:** Cost-Benefit Analysis, Stable Angina, Coronary Artery Disease, Computed Tomography Angiography, Unified Health System, Diagnostic Imaging/methods

In the evaluation of patients with stable coronary artery disease (CAD), also called chronic coronary syndrome (CCS), complementary tests are used both for diagnostic and prognostic purposes.^1 , 2^ Anatomical (coronary angiography and coronary CT angiography) and functional (exercise testing, stress echocardiography, rest and stress myocardial perfusion imaging by scintigraphy, magnetic resonance, and positron emission tomography) tests are available. Coronary angiography, the gold standard, is invasive and therefore, indicated for clinically more severe patients, or those with poor prognostic findings in non-invasive testing, when myocardial revascularization is considered or planned.^3^

The choice for the most appropriate diagnostic test is an important and challenging issue for the cardiologist in the clinical evaluation of CCS. The first step in this decision-making process is the assessment of the pre-test probability (PTP) of CAD. As recommended by the current SCC guidelines,^3 , 4^ patients classified as having high PTP should receive medical therapy and undergo testing for prognostic information. The patients with low PTP should be assessed for an alternative diagnosis more likely than CAD. Patients with PTP calculated between 15-85% are in the intermediate range, where the complementary tests are more useful and important for CAD diagnosis.^5^ In addition to diagnostic accuracy and PTP, the selection of a non-invasive test depends on the clinical characteristics of patients, local expertise, and the availability of tests.^4^ In Brazil, it is estimated that up to 80% of the population depends exclusively on medical care provided by the public health system (SUS).^6^ In this context of managing economic resources, physicians and health managers should focus on the most cost-effective options for the diagnosis of CAD.

The article by Carmo et al.^7^ assesses just the intermediate and low-intermediate PTP (10-60%) scenario, via two different methods of cost-effectiveness analysis, using up-to-date concepts of health technologies (incremental cost-effectiveness ratio and the net benefit). The strategies with sequential tests were performed when the first test was positive. The results were presented according to the variation of PTP at different thresholds of willingness to pay for a correct diagnosis. Although coronary computed tomography angiography (CTA) is not yet available in SUS, it was the most cost-effective strategy in this study, either alone or in sequential testing, except in the lower thresholds of willingness to pay, in which was overwhelmed by stress echocardiogram (SE).^7^

Another interesting finding concerns the use of exercise testing (ET), which was placed in the background in international guidelines,^4^ but showed to be an excellent cost-effective option in lower PTPs and lower willingness to pay thresholds, especially when followed by SE in case the ET was positive.^7^ Given the large economic differences between regions in Brazil, in locations with less availability of resources and health financing, ET could remain the main diagnostic screening strategy for CAD.

Myocardial scintigraphy (MS), widely used in SUS, proved to be more expensive and less effective than CTA and SE in all scenarios evaluated, appearing as a negative spotlight in the diagnostic strategy in CAD. Furthermore, CTA was able to reveal non-obstructive CAD even in patients with moderate and severe myocardial ischemia in functional tests, such as 15% of those initially selected for the ISCHEMIA trial.^8^ Another advantage of CTA is the possibility of non-invasive quantification of the fractional flow reserve, capable of detecting flow-limiting obstructive coronary lesions, reducing the number of false-positive results.^9^ These findings highlight the usefulness of CTA in significantly reducing the number of CCS patients referred to coronary angiography and, therefore, decreasing the costs and possible complications of the invasive testing.

The main results of the analysis performed by the authors are based on the estimated CTA price in which SUS would pay for, that can be underestimated, since the ATC versus MS costs readily available online in many supplementary healthy services are comparable. This would lead to an important limitation of this manuscript if confirmed afterwards. Another gap is the intermediate-high PTP scenario (60-85%), not evaluated in this study, where MS could be able to show a better competitiveness, considering its good performance in confirming the diagnosis of functionally significant CAD in this higher PTP range.^10^

This article shows relevant evidence that may be applied by SUS health managers and physicians in the decision-making process of the diagnostic methods chosen for CAD. It might also be used as a future reference for local guidelines, that similarly to other international guidelines^1 , 11^ may consider the recommendation of ATC as a first-line diagnostic test for CAD, as an alternative to functional imaging. It should be noted, however, that functional testing remains irreplaceable in objectively assessing the degree of functional limitation and the patient’s response to therapy.^3 , 4^ Finally, there is still room for the rational use of all available methods in diagnosing obstructive CAD in clinical practice, but there is no longer any reason why CTA should not be incorporated into the SUS.
